# Supplementary material for: Splice-Junction-Based Mapping of Alternative Isoforms in the Human Proteome
Source: Cell Rep. Author manuscript; Available in PMC 2020 Jan 15. (PMC6961840; doi:10.1016/j.celrep.2019.11.026)
Supplement: 3 [file NIHMS1546469-supplement-3.zip › DF2/PXD0010154/Liver-564-O75369-FGGVDIPNSPFTVMATDGEVTAVEEAPVTEEAYVP.pdf]

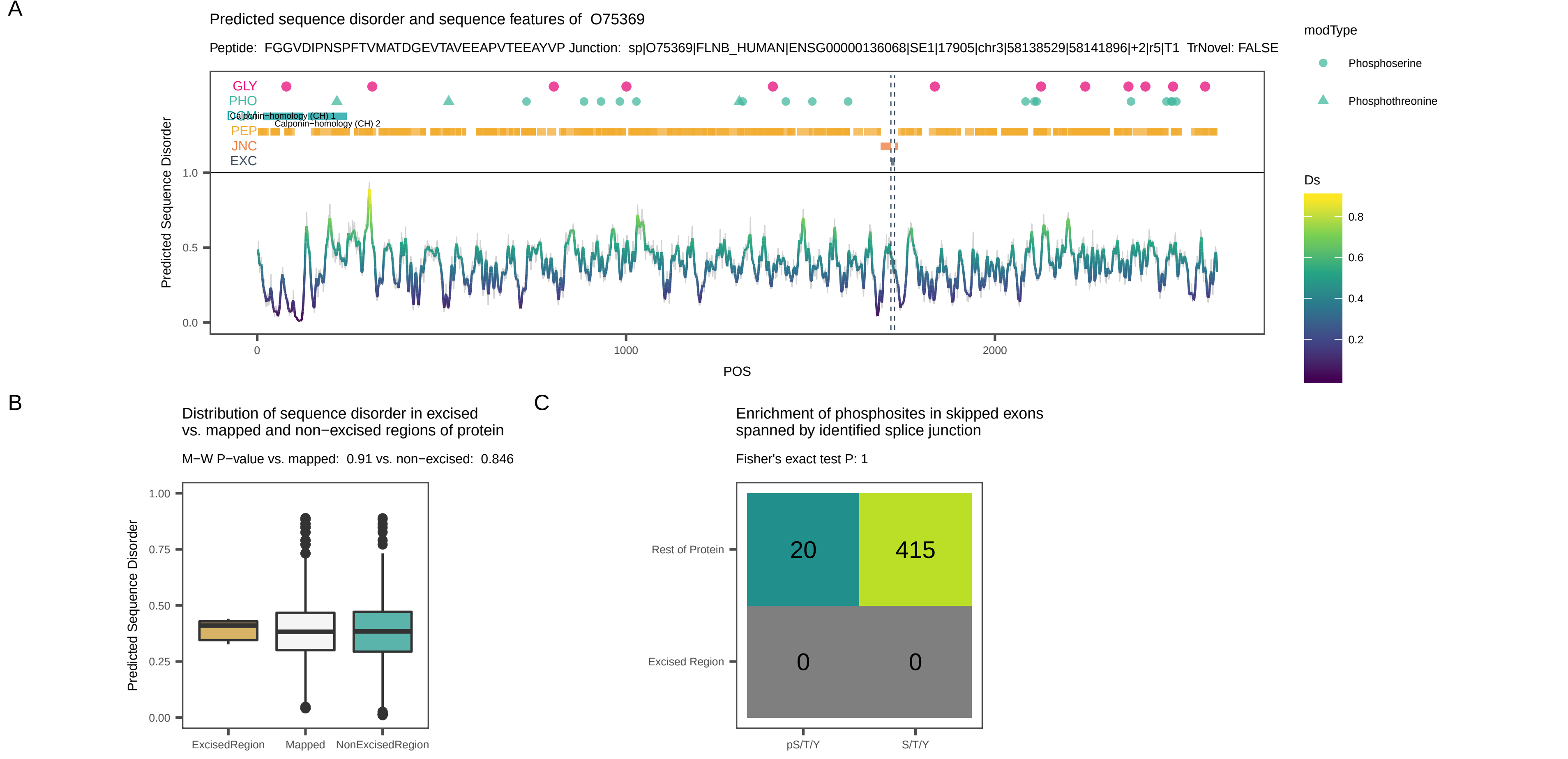
A
Predicted sequence disorder and sequence features of O75369
Peptide: FGGVDIPNSPFTVMATDGEVTAVEEAPVTEEAYVP Junction: sp|O75369|FLNB_HUMAN|ENSG00000136068|SE1|17905|chr3|58138529|58141896|+2|r5|T1 TrNovel: FALSE
GLY
PHO
Calponin−homology (CH) 1
Calponin−homology (CH) 2
PEP
JNC
EXC
Predicted Sequence Disorder
1.0
0.5
0.0
0
1000
2000
POS
modType
Phosphoserine
Phosphothreonine
Ds
0.8
0.6
0.4
0.2
B
Distribution of sequence disorder in excised
vs. mapped and non−excised regions of protein
M−W P−value vs. mapped: 0.91 vs. non−excised: 0.846
Predicted Sequence Disorder
1.00
0.75
0.50
0.25
0.00
ExcisedRegion
Mapped
NonExcisedRegion
C
Enrichment of phosphosites in skipped exons
spanned by identified splice junction
Fisher's exact test P: 1
Rest of Protein
Excised Region
20
415
0
0
pS/T/Y
S/T/Y
